# Renal Epithelioid Angiomyolipoma: A Case Report

**DOI:** 10.1002/ccr3.71440

**Published:** 2025-11-09

**Authors:** Bahareh Mehramouz, Negin Frounchi, Sepideh Hadimaleki, Parisa Mehrasa

**Affiliations:** ^1^ Department of Pathology, Imam Reza Hospital Tabriz University of Medical Sciences Tabriz Iran; ^2^ Kidney Research Center Tabriz University of Medical Sciences Tabriz Iran; ^3^ Department of Pathology Tabriz University of Medical Sciences Tabriz Iran

**Keywords:** angiomyolipoma, case report, kidney neoplasms, nephrectomy, PEComas, renal tumor

## Abstract

Angiomyolipoma (AML) is a rare benign mesenchymal tumor, constituting less than 10% of renal masses, and occurs sporadically or in association with tuberous sclerosis complex (TSC). AML is generally categorized into classic AML and epithelioid angiomyolipoma (EAML), with the latter comprising approximately 4.6% of AML cases. EAML is characterized by a predominance of epithelioid cells, minimal adipose tissue, and distinct histological features such as necrosis and potential malignancy, which can lead to misdiagnosis as renal cell carcinoma (RCC). This report presents a case of renal EAML in a 27‐year‐old Iranian woman with mild left flank pain and hematuria, with imaging studies revealing an echogenic mass in the left kidney suggestive of AML. Contrast‐enhanced computed tomography and magnetic resonance imaging confirmed a heterogeneous mass with fat content. Histopathological examination post‐partial nephrectomy revealed a carcinoma‐like lesion with a proliferation of epithelioid cells, necrosis, and scattered giant cells, consistent with EAML. Notably, the tumor exhibited low mitotic activity and lacked atypical mitoses, confirming its benign nature. Despite the rarity of EAML and the lack of distinct clinical or radiological features, this case underscores the importance of pathological findings for accurate diagnosis. The management of EAML remains challenging due to its aggressive potential; however, surgical intervention is often the gold standard treatment. This case contributes to the limited literature on EAML, emphasizing the need for further research to establish definitive diagnostic criteria and treatment guidelines to address its clinical uncertainties.

**Summary**

- Renal epithelioid angiomyolipoma, a rare, potentially malignant tumor resembling renal cell carcinoma, requires histopathological and immunohistochemical analysis for diagnosis.
- Surgical resection, such as partial nephrectomy in small tumors, remains the cornerstone of treatment for localized tumors, with careful postoperative monitoring to detect recurrence or metastasis.

## 1 Introduction

Angiomyolipoma (AML) is a rare benign mesenchymal mass that constitutes < 10% of all renal masses [1, 2]. Renal AMLs are classified into two categories: classic AML and epithelioid AML (EAML). The classic AML is a benign lesion often discovered incidentally on magnetic resonance imaging (MRI) or computed tomography (CT) scans due to the presence of a fat component and the absence of calcification. It comprises a variable amount of triphasic histology including thick‐walled blood vessels, mature adipose tissue, and smooth muscle tissue [3]. EAML is an extremely rare subtype of AML, accounting for about 4.6% of all diagnosed AML cases [4]. The highest incidence of EAML is in middle‐aged females [5]. It is predominantly composed of epithelioid cells (at least 80%) with minimal adipose tissue.

Additionally, EAML is distinct from the classic variant due to its histological features including necrosis, potential malignancy, and the possibility of lymph node and distant metastases [6, 7]. Due to the histological variability of neoplastic cells and the presence of multinucleated and bizarre cells, EAML may be misdiagnosed as other malignant tumors such as renal cell carcinoma [1]. Previous studies suggest that immunohistochemical markers like HMB‐45 can differentiate EAML from other tumors such as sarcomas [8]. Despite numerous case reports on EAML, there are no specific clinical or radiological features unique to this tumor. Therefore, pathological findings remain the primary tool for diagnosis [5, 8]. Here, we present a rare instance of renal EAML in a patient without TSC, emphasizing the diagnostic approach, management strategies, and clinical outcomes. The rarity of this condition and its potential for malignancy underscore the importance of this report in contributing to the existing literature and guiding clinical practice.

## 2 Case Presentation

A 27‐year‐old Persian woman with no contributory significant medical, drug or familial history presented to the clinic with mild left flank pain and gross hematuria. She denied NSAID use or any other medications. The patient reported intermittent flank pain persisting for 1 year (without functional impairment) accompanied by episodes of hematuria in the past month. The clinical examination revealed no significant findings; there was no flank tenderness, her vital signs remained stable, with a blood pressure of 110/70 mmHg, a respiratory rate of 22 breaths per minute, a pulse rate of 77 beats per minute, and a body temperature of 36°C.

Routine blood tests at the visit time showed a slightly elevated white blood cell count of 12,900 × 1000/mm^3^. The red blood cell count was 4.46 million/mm^3^, hemoglobin levels were 12.5 mg/dL, and platelet count was 318,000 × 1000/mm. Platelets (318,000 × 10^3^/mm^3^), electrolytes (potassium 4.6 mmol/L, sodium 142 mmol/L), and renal function markers (urea 28 mg/dL, creatinine 0.8 mg/dL) were unremarkable. Liver enzymes (aspartate aminotransferase 21 U/L, alanine aminotransferase 30 U/L, alkaline phosphatase 219 U/L) and total bilirubin (0.57 mg/dL) were in expected ranges. In contrast, the urine analysis showed reddish, turbid urine with acidic pH (5), +3 protein, +3 hemoglobin, numerous white blood cells, many red blood cells, and high bacterial counts. Glucose, ketones, nitrites, and crystals were absent.

**FIGURE 1:**
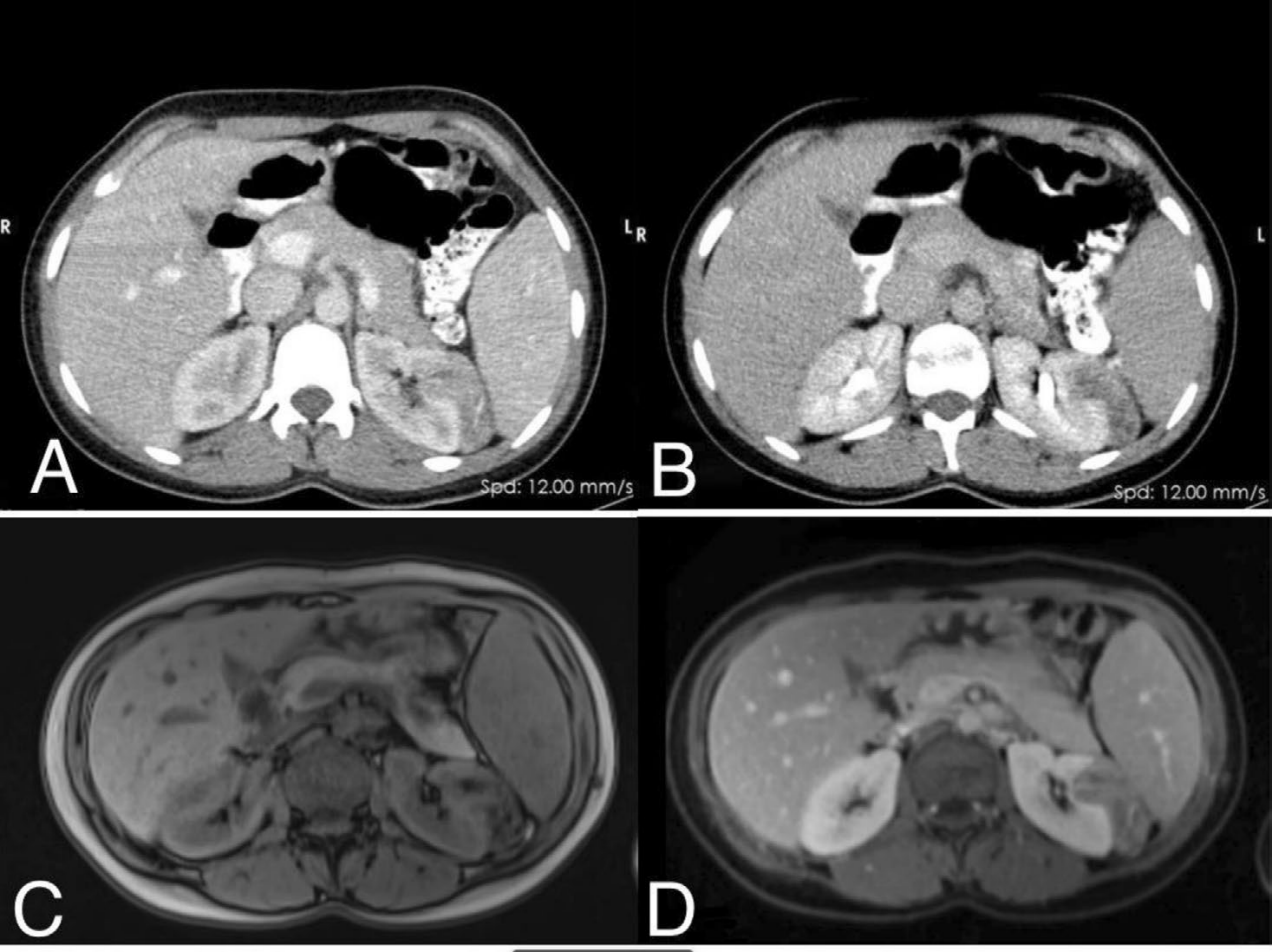
(A, B) Axial CT scan showing an irregularly shaped, hypodense mass with a heterogeneous pattern in the left kidney (A: Arterial phase, B: Delayed phase). (C, D) Axial T1‐weighted MRI demonstrating a lobulated, heterogeneous mass with fat content in the mi d‐pole of the left kidney.

Abdominal ultrasonography demonstrated an echogenic mass with well‐defined margins, measuring 32 × 28 mm, in the upper pole of the left kidney, suggestive of an angiomyolipoma. Both kidneys exhibited normal size and echogenicity. However, Grade III hydronephrosis was noted in both kidneys, with a pelvic diameter of 16.5 mm and 20 mm in the right and left kidney, respectively, indicating potential ureteropelvic junction (UPJ) obstruction. No renal calculi were identified. Subsequent contrast‐enhanced computed tomography, revealed an irregularly shaped, heterogeneous mass with a chest wall defect (likely due to invasion by the mass) in the upper pole of the left kidney. The mass measured 49 × 46 × 10 mm and had mild enhancement post‐contrast, raising the possibility of an AML. Further magnetic resonance imaging showed a well‐defined lobulated mass measuring 53 × 41 × 34 mm in the mid‐pole of the left kidney. It exhibited a heterogeneous signal and exophytic extension. Fat‐suppression sequence showed a signal drop, suggesting fat content within the lesion. During the contrast phase, heterogeneous enhancement of the mass was noted, providing additional evidence suggestive of AML (Figure 1).

### 2.1 Differential Diagnosis, Investigation and Treatment

**FIGURE 2:**
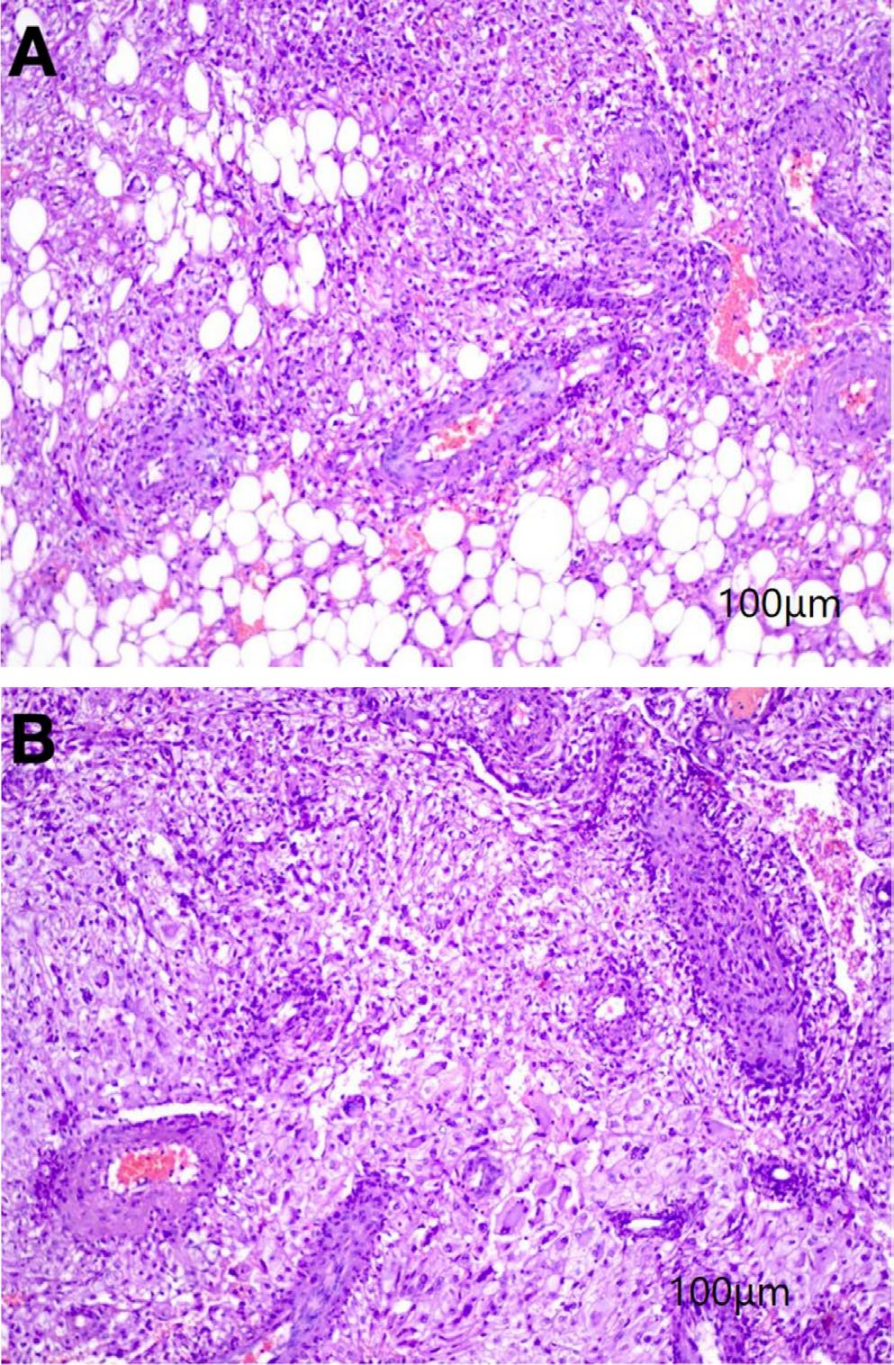
(A, B) Sections show kidney tissue with carcinoma like humoral lesion, composed of proliferation of polygonal cells with abundant pink to focally clear cytoplasm and some atypic in nuclei, admixed with fatty tissue and thick‐walled vessels. Some mitotic activity, necrosis, hemorrhage, scattered giant cells and intranuclear inclusion body also are detected.

Two weeks later the patient underwent partial nephrectomy for histological confirmation and definitive treatment of an exophytic kidney mass. Gross examination demonstrated a brown‐colored tumor with peripheral fat. Peripheral fat was free and capsular invasion was not seen. The largest diameter of the tumor was 5 cm. Histopathological examination (Figure 2) of the nephrectomy specimen revealed a carcinoma‐like lesion composed of a proliferation of polygonal cells with abundant pink to focally clear cytoplasm, some nuclear atypia (Grade II), and an admixture of fatty tissue and thick‐walled vessels. Remarkable findings included mitotic activity, necrosis, hemorrhage, scattered giant cells, and intranuclear inclusion bodies. These histological results were consistent with epithelioid angiomyolipoma, with 100% of the neoplastic cells displaying an epithelioid phenotype. The mitotic count was less than 2 per 10 high‐power fields with no atypical mitoses.

Imaging and pathology findings suggested AML, leading to immunohistochemical staining to rule out clear cell carcinoma. Hematoxylin and eosin stains and paraffin‐embedded block analysis demonstrated the proliferation of spindle and epithelioid cells mixed with fat and some thick‐walled vessels. The staining results were positive for SMA and HMWB45, while negative for PAX8, CKAE1/AE3 supporting the diagnosis of epithelioid angiomyolipoma.

### 2.2 Outcome and Follow‐Up

**FIGURE 3:**
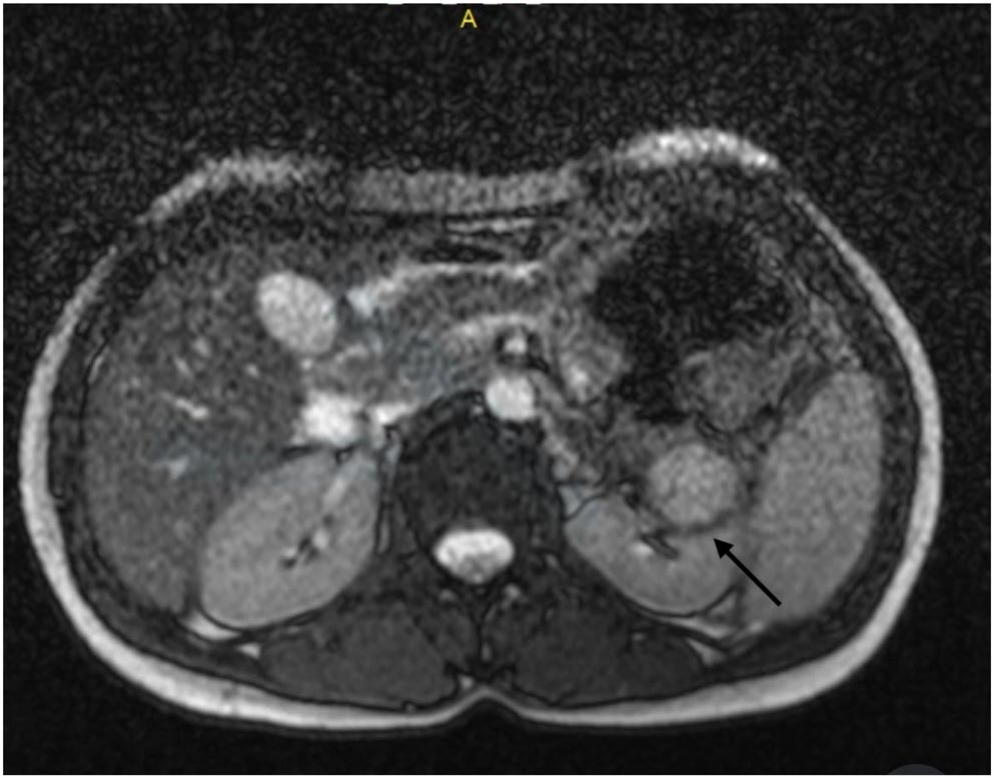
Axial T2‐weighted MRI image of the kidney post‐partial nephrectomy, performed following the occurrence of hematuria. The image reveals evidence of inflammation and hematoma formation.

In this case, the tumor showed 100% atypical epithelioid cells and necrosis, meeting two of the four criteria for risk stratification of epithelioid angiomyolipoma (EAML), which include (a) > 70% atypical epithelioid cells, (b) > 2 mitotic figures per 10 high‐power fields (HPF), (c) atypical mitoses, and (d) necrosis [9, 10]. The presence of two major risk factors classified the tumor as intermediate risk. Following the surgical procedure, the patient's flank pain resolved. However, multiple episodes of hematuria recurred up to 3 months after surgery, which subsequently resolved without intervention (Figure 3).

We used follow‐up protocol aligns with renal masses and localized renal cancer, as outlined in the American Urological Association (AUA) guidelines [11]. The patient underwent periodic physical examination and laboratory testings. The follow‐up CT‐scan 6 months after the surgery showed no evidence of mass or cyst formation and metastasis, both kidneys appeared normal this result shows effective management of the EAML, with no sign of recurrence or metastasis during the final follow‐up.

## 3 Discussion

EAML is an exceptionally aggressive and rare variant of AML, accounting for approximately 0.3%–3.0% of the general population and 1% of surgically resected renal masses [2]. Unlike classic AML, which is usually benign and associated with Tuberous Sclerosis Complex (TSC), EAML demonstrates malignant potential including local recurrence post‐resection and distant metastasis [4, 5].

Our case highlights the therapeutic challenges of managing EAML. While most classical types of AML patients are asymptomatic [7], EAML frequently presents with symptomatic features such as flank pain or hematuria, mirroring the clinical profile of classic AML [12].

This case presented with mild left flank pain and was found to have a renal mass initially suspected to be a conventional AML or malignancy. EAML is often compared to renal cell carcinoma due to its atypical vascular features and imaging characteristics. On CT scans EAML typically presents as a hyperdense lesion with early and late wash‐out, internal hemorrhage, and heterogeneity. However, imaging findings are often non‐specific, making differentiation from RCC or fat‐poor AML challenging [5].

In our case report, abnormalities in the kidneys suggested angiomyolipoma, but imaging revealed a well‐defined mass with lobulated margins and exophytic extension, raising suspicion for malignancy. Definitive diagnosis of EAML needs both immunohistochemical (IHC) and pathological examination, which reveals epithelioid cells with polygonal or round shapes, clear or eosinophilic cytoplasm, and Grade II of nuclear atypia and mitotic activity.

Because EAML is uncommon and has unpredictable clinical behavior, its management is still debatable. Surgical resection is the cornerstone of treatment, with partial nephrectomy preferred for small, localized tumors to maintain renal function [13, 14]. However, vigilant monitoring is critical due to the risk of metastasis and recurrence. In this instance, the patient showed no signs of recurrence at the 12‐month follow‐up, in line with the favorable results for localized EAML documented in previous literature [15, 16, 17].

This case emphasizes how crucial it is to take EAML into account when making a differential diagnosis of renal masses, especially in patients without TSC. Given its rarity and aggressive potential, further research is essential to establish standardized diagnostic and therapeutic guidelines [6, 8].

## Author Contributions

**Bahareh Mehramouz:** conceptualization, data curation, supervision, writing – review and editing. **Negin Frounchi:** data curation, investigation, methodology, writing – original draft. **Sepideh Hadimaleki:** conceptualization, data curation, methodology, writing – original draft. **Parisa Mehrasa:** conceptualization, supervision, writing – review and editing.

## Consent

Written informed consent was obtained from the patient for publication of this case report and any accompanying images.

## Conflicts of Interest

The authors declare no conflicts of interest.

## Data Availability

All data generated during this study are included in this published article.
